# Practical Chemistry and Pharmacy

**Published:** 1894-02

**Authors:** 

**Affiliations:** LaGrange, Ga.


### PRACTICAL CHEMISTRY AND PHARMACY.

EDITED BY HENRY R. SLACK, M. D., PH. M., LAGRANGE, GA.

### SACCHARATED PEPSIN.

The United States Pharmacopœia of 1880 defines saccharated pepsin as: "Pepsin, the digestive principle of the gastric juice, obtained from the nervous membrane of the stomach of the hog mixed with powdered sugar of milk." No proportions are given, as no standard was fixed for the pepsin employed, the test being made with the saccharated article which is required to digest at least fifty times its weight of coagulated egg albumen, under the condition laid down in the book.

The Pharmacopœia of 1890, which went into effect January 1st of this year: Pepsin is made official, its digesting power being fixed at not less than 1 to 3,000, under the conditions prescribed, which differ somewhat from those set down in the edition of 1880. The new official saccharated pepsin is made by triturating ten grains of the above-mentioned pepsin with ninety grains of milk sugar recently dried. It will thus be seen that our saccharated pepsin of 1890 will have about six times the digestive power of the old, and it is important that we should use the best. —*D. C. and Chem. Gaz.*

---

### MICRO-CHEMICAL TEST FOR TUMERIC.

Hans M. Wilder, Ph. G., accidentally found, while experimenting, a test for the presence of tumeric in powdered rhubarb and yellow mustard, which he has often used and found reliable. This test is based on the fact that volatile oils (sassafras, fennel, anise, etc.) dissolve the coloring matter of tumeric, while they do not extract any coloring matter from either rhubarb or mustard. The method of application is very simple. Mix a little of the suspected powder with any volatile oil, and examine under the microscope. The smallest speck of tumeric present will be found surrounded by a yellow zone, while the particles of rhubarb or mustard will merely have their color brightened. In absence of tumeric the microscopic field will remain colorless, while tumeric will instantly color the whole or part of the field yellow.—*Drug. Circ.*

---

### MIXING TURPENTINE WITH ACID.

In mixing turpentine with a strong acid, or with tr. iodine, the latter should always be added to the former, for a violent reaction occurs between them. Such a mixture should never be made in a bottle or similar vessel, but in an open dish or mortar, and the acid should only be added in small portions at a time. When the operation is begun the inexperienced operator may be deceived as to the reaction, as it does not begin immediately. He should wait until effervescence ensues; then as the mixture becomes warm from chemical action the results of adding new portions will show themselves more quickly. After the acid is all in, the mixture should be well stirred with a glass rod allowed to remain in the open dish for some time, and, finally, when transferred to the bottle, the latter should be left uncorked for some time.

---

### COMPOUND EXTRACT SALIX.

Doubtless many of our readers have been asked the question, "Doctor, what is Compound Extract of Salix, used to preserve fruit?" The answer should be, "Nothing but *ordinary salicylic acid* which you can buy from your druggist at half the price asked for the advertised stuff." We analyzed this substance as soon as it came out and found it to be nothing but salicylic acid. Dr. Payne, the State chemist, and the United States Government chemist have also reported analyses with the same result.

---

### POISONOUS LINSEED OIL.

An examination was lately made by Pieszceck, of some linseed oil, which, when taken into the stomach, produced general indisposition, dizziness and vomiting, but nothing could be discovered to account for the strange phenomena. A sample, however, of the linseed which had yielded the oil was found upon analysis to contain not less than thirty-five per cent. of impurities, including especially fifteen per cent. of the seeds of darnel, a common meadow weed.—*Apoth. Zeit.*

---

### EFFECT OF RHUBARB ON THE URINE.

According to Jung if the urine of a healthy person who has been taking rhubarb be tested with bismuth, the same brown coloration will be produced as if sugar were present. Inquiry should be therefore made respecting the use of the first named drug, in cases where it might otherwise lead to a wrong diagnosis. (Druggists' Circular.) This is of special interest to medical examiners of life insurance.

